# Dental Disorders and Salivary Changes in Patients with Laryngopharyngeal Reflux

**DOI:** 10.3390/diagnostics12010153

**Published:** 2022-01-09

**Authors:** Sabrina Casciaro, Matteo Gelardi, Rossana Giancaspro, Vitaliano Nicola Quaranta, Giuseppe Porro, Brigida Sterlicchio, Antonia Abbinante, Massimo Corsalini

**Affiliations:** 1 Dental Hygienist, 74018 Palagianello, Italy; sabrina.casciaro@outlook.it; 2 Unit of Otolaryngology, Department of Clinical and Experimental Medicine, University of Foggia, 71121 Foggia, Italy; matteo.gelardi@unifg.it; 3 Department of Basic Medical Sciences, Neurosciences and Sense Organs—Section of Respiratory Disease, University of Bari Aldo Moro, 70124 Bari, Italy; vitalianonicola.40@gmail.com; 4 Unit of Otolaryngology, Hospital of Lecce, 73100 Lecce, Italy; giuseppeporro1@gmail.com; 5 ENT Specialist, 76123 Andria, Italy; brigidasterlicchio87@gmail.com; 6 Dental Hygienic Studies Course, University of Bari, 70125 Bari, Italy; presidente@aiditalia.it; 7 Department of Interdisciplinary Medicine, University of Bari, 70124 Bari, Italy; massimo.corsalini@uniba.it

**Keywords:** laryngopharyngeal reflux, dental disorders, salivary flow, salivary Ph, pepsin, PEP test

## Abstract

**Background:** Laryngopharyngeal reflux (LPR) is a common inflammatory condition of the upper aerodigestive tract tissues related to the effects of gastroduodenal content reflux, characterized by a wide variety of clinical manifestations. The aim of our study was to evaluate the possible association between dental disorders and LRP, focusing on the role of salivary changes. **Methods**: Patient’s dental status was evaluated according to Schiff Index Sensitivity Scale (SISS), Basic Erosive Wear Examination (BEWE) and Decayed, Missing, and Filled Teeth (DMFT) scores. Reflux-associated symptoms were assessed according to Reflux symptom index (RSI). A qualitative and quantitative examination of saliva was performed. **Results**: Patients suffering from LPR had a higher incidence of dental disorders, regardless the presence of salivary pepsin, and thus, statistically significant higher scores of RSI (*p* = 0.0001), SISS (*p* = 0.001), BEWE (*p* < 0.001) and VAS (*p* < 0.001). Moreover, they had lower salivary flow compared with healthy patients. **Conclusions**: The finding of demineralization and dental caries on intraoral evaluation must raise the suspicion of LRP. Reflux treatments should also be aimed at correcting salivary alterations, in order to preserve the buffering capacity and salivary pH, thus preventing mucosal and dental damage.

## 1. Introduction

Laryngopharyngeal reflux (LPR) is a common inflammatory condition of the upper aerodigestive tract tissues related to both direct and indirect effects of gastroduodenal content reflux [1]. The direct action of gastric contents, acid, pepsin and conjugated bile acids on pharynx and larynx can lead to morphological changes in the upper aero-digestive region, including supraglottic edema and erythema, posterior pharyngeal wall cobblestoning, vocal cord ulcers, interarytenoid changes, medial arytenoids wall edema and vocal cord granulomas [2]. Due to these anatomical changes, LPR-associated symptoms embrace a wide variety of clinical manifestations, such as cough, chronic repetitive throat clearing, throat pain, hoarseness, excessive mucus production, chronic cough, dysphagia and globus pharyngeus [3]. LPR diagnosis is commonly made by primary care physicians, otolaryngologists, allergists and gastroenterologists combining patients’ medical history and clinical evaluation with endoscopic findings, esophageal impedance-pH monitoring, salivary PEP test and/or patient-reported outcomes measures, such as the reflux symptom index (RSI) [4,5]. The clinical evaluation of patients who complain of symptoms related to LPR also consists of a careful intraoral examination; thus, the assessment of the dental status could be useful in the diagnostic process. As a matter of fact, the correlation between GER and dental erosion, erythema of the oral cavity, oral ulcers, gingivitis, periodontitis and glossitis has already been demonstrated [6]. Since LPR is often considered a substantially rare manifestation of GER disease, it has been questioned whether LPR was also associated with dental disorders such as dental erosions and caries, which may unveil the disease, allowing for an earlier diagnosis and, consequently, a better prognosis [7].

Based on this background, the aim of our study was to evaluate the correlation between dental status and LRP, focusing on the potential role of salivary changes in the pathogenesis of dental disorders and on the possibility of using treatments aimed at preserving salivary homeostasis in patients with reflux to prevent dental demineralization.

## 2. Materials and Methods

An observational study was conducted enrolling 30 consecutive patients who complained of signs and symptoms referable to gastroesophageal reflux (GER) and LPR at the Department of Otolaryngology of the University Hospital of Bari. The age of the patients ranged from 19 to 69 years (mean 40.6). Specific exclusion criteria were age <18 years old, diabetes, smoking and alcoholic habit, malignancies and ongoing pharmacological treatments. Informed written consent was obtained from all participants. The study was approved by the Area 1 Ethics Committee of the University Hospital of Bari.

### 2.1. Data Collection

The medical history of each patient was carefully evaluated, paying greater attention to the patients’ socio-economic status, eating habits, oral hygiene habits, intake of drugs that could cause dental erosions and presence of extra-esophageal symptoms of GER. All study subjects were assessed for gastrointestinal symptoms by using Reflux symptom index (RSI), a nine-item self-administered outcome questionnaire, which assesses the symptoms of laryngopharyngeal reflux and their severity [8] (Table 1). A total RSI score of more than 13 allowed for a clinical diagnosis of LPR. According to the RSI score, patients were divided into two groups. The experimental group (n = 20) consisted of patients diagnosed with LPR, while the control group (n = 10) consisted of patients with an RSI score <13, non-diagnostic for LPR. 

### 2.2. Clinical Examination

All patients underwent a thorough intraoral examination to evaluate any signs and symptoms, including dental erosion, aphthous lesions, erythema of the soft palate and uvula, glossitis and mucous lesions. Moreover, they underwent transnasal laryngopharyngoscopy, performed with a 3.4 mm diameter flexible fiberscope (Vision-Sciences^®^ ENT-2000), to confirm or exclude the diagnosis of LPR. Dentin hypersensitivity was assessed after an air blast test by both the examiner and the patient. In particular, the examiner assigned a score according to the Schiff Index Sensitivity Scale (SISS), which evaluates the degree of DH as a function of the patient stimulus reaction (0 = no response; 1 = response to the stimulus, not requiring withdrawal of stimulus; 2 = response to stimulus, requiring withdrawal of stimulus; 3 = response to stimulus, which is considered painful, requiring discontinuation of stimulus). Furthermore, patients had to assign a score to the stimulus, according the VAS scale, ranging from 0 (absence of pain) to 10 (maximum pain) [9]. Tooth wear was also evaluated according to the Basic Erosive Wear Examination (BEWE), which assesses the damage according to the tooth-affected surface regardless of its depth in dentin. In particular, each tooth, except the third molars, were examined on the five faces and scored from 0 to 3 (0 = no erosive tooth wear; 1 = initial loss of surface texture; 2 = distinct defect, hard tissue loss <50% of the surface area with possible exposure of dentine; 3 = hard tissue loss ≥50% of the surface area with systematic exposure of dentine). Each arch was divided into three sextants. The total BEWE index, ranging from 0–18, was obtained from the sum of the highest score for each sextant [10,11]. The dental caries status of each patient was scored according to the Decayed, Missing and Filled Teeth (DMFT) index. Teeth were classified as decayed (DT) if there was evidence of cavitation of the crown or root, missing (MT), if absent, or filled (FT), if without secondary caries. The total DMFT score was given by the sum of DTs, MTs and FTs.

### 2.3. Qualitative and Quantitative Examination of Saliva

To determine salivary pH, patients were asked to rinse their mouths with 50 mL of distilled water and then collect their saliva in sterile tubes. The salivary pH of each patient was assessed immediately after collection using litmus paper, which was immersed into the tube containing the collected saliva for 1 or 2 s and interpreted after 5 s according to the color table, where red indicates an acid solution, green a neutral solution and blue an alkaline solution [12]. Moreover, to determine the unstimulated salivary flow rate (uSFR), saliva samples were collected for 15 min into calibrated dry plastic tube, at least one hour after the last intake of food and beverages. The flow rate was calculated in milliliters per minute (mL/min). In particular, a uSFR <0.1 mL/min was considered indicative of hyposalivation [13,14]. The salivary samples were also analyzed using a PEP test kit immediately after the salivary collection in order to determine the salivary pepsin concentration. The test required 1 mL of salivary sample with the addition of 1 mL of 0.01 M citric acid. Each sample was centrifuged at 400 rpm for 5 min at normal room temperature. Subsequently, 80 μL of supernatant was added to 240 μL of migration buffer and then vortexed for 10 s: 80 μL. Results were ready after 15 min. In particular, the T band revealed the pepsin presence, whose quantity was directly proportional to the intensity of the T band, while the C band estimated the integrity of the system [15]. The pepsin concentration levels were accurately measured using an electronic reader, with a detection limit of 16 ng/mL and a quantitation range between 25 ng/mL and 500 ng/mL.

### 2.4. Statistical Analysis

The Kolmogorov–Smirnov test was used to evaluate the normal distribution of data. Categorical values were analyzed using the chi-square test or Fisher’s exact test as appropriate and were reported as n (%). Continuous variables were compared by Student’s *t*-test for independent samples for normally distributed data or by Mann–Whitney U test for non-normally distributed data. Continuous parameters with normal distribution are reported as mean ± standard deviation, while those without normal distribution are reported as median (interquartile range). A linear correlation test was performed between the Pepsin values and the RSI values. Univariate binomial logistic regression analysis was performed to define membership in the group of patients with laryngeal esophageal reflux. A ROC curve was constructed to define the accuracy of belonging to the LPR group starting from the salivary flow. To correctly classify the LPR group with the control group, linear discriminant analysis was used using two variables of major clinical interest, salivary flow and salivary pH. To classify cases into categorical divisions, linear canonical discriminant analysis was used to create a model that optimizes distances between sample classes and within sample classes. The percentage of cross-validated accuracy (CVA, %) was calculated using the “leave-one-out method” to provide a percentage that reflects the amount of agreement between clinical and model-based classification. The level of statistical significance was set at *p*-value < 0.05. All statistical analyses were performed using SPSS for Windows 23.0 (SPSS, Chicago, IL, USA).

## 3. Results

Recruited patients were divided into two groups according to the RSI score. The experimental group (Group A, n = 20) consisted of patients suffering from LPR, with an average RSI score of 23.35, while the control group (Group B, n = 10) consisted of patients with an RSI score <13 (mean RSI = 3.6), non-diagnostic for LPR. The endoscopic examination of the upper aerodigestive tracts confirmed the diagnosis of LPR in the Group A patients and excluded the aforementioned diagnosis in Group B patients. 

### 3.1. Intraoral Manifestations

The intraoral examination of Group A patients revealed dental erosions in 15 (75%) patients, including 13 (86.7%) patients with second degree erosions and two (13.3%) with third degree erosions, glossitis in seven (35%) patients; aphthosis in four (20%) patients and hard and soft palate erythema and edematous uvula in 18 (90%) patients. Reported symptoms were hyperesthesia (n = 16, 80%), halitosis (n = 11, 55%), dysgeusia (n = 9, 45%), xerostomia (n = 14, 70%) and burning mouth (n = 4, 20%). The mean DMFT index of Group A was 6.45. The intraoral examination of Group B patients revealed dental erosions of first degree in one (5%) patient, xerostomia in two (10%) patients, halitosis in one (5%) patient and hyperesthesia in three (15%) patients. The mean DMFT index of Group B was 4. 

### 3.2. Salivary Pepsin Levels and Related Symptoms

**Figure 1:**
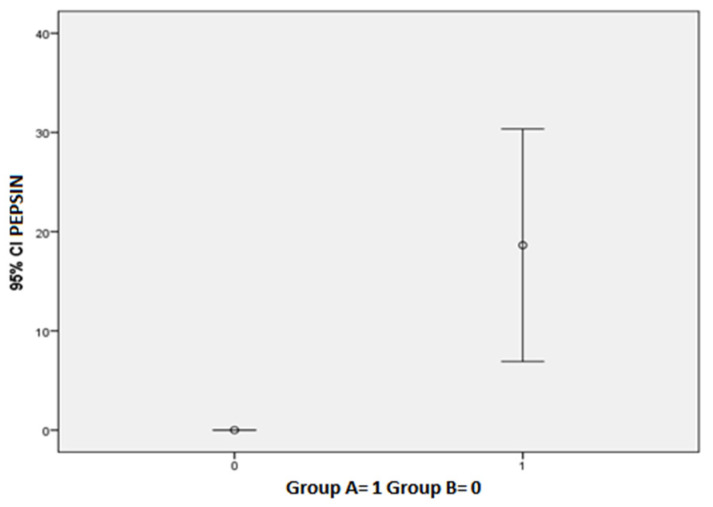
Group A patients showed a statistically significant higher value of Pepsin (*p* = 0.027).

**Figure 2:**
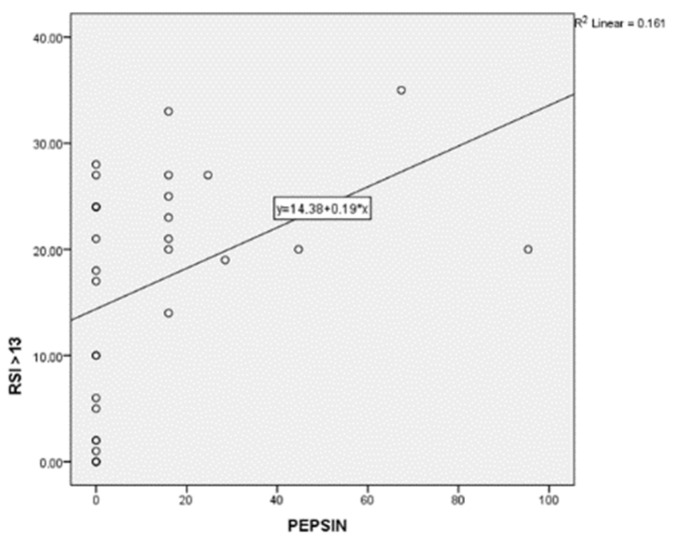
Oral pepsin levels correlated linearly with RSI scores (*p* = 0.028).

Twelve (60%) Group A patients tested positive for PEP Test (subgroup A PEP +), while eight (4%) patients tested negative (subgroup A PEP −). Among the PEP test-positive patients, all of them had cavity erythema, nine (45%) dental erosions (including seven patients with second degree erosions and two patients with third degree erosions), four (20%) glossitis and four (20%) aphthosis. The mean DMFT index of subgroup A PEP + was 5.5. The mean DMFT index of subgroup A PEP was 7.8. PEP Test positive patients complained of more oral manifestations than PEP test negative patients, such as hyperesthesia, dysgeusia, xerostomia, sialorrhea and burning mouth. Group A patients showed a statistically significant higher value of Pepsin (18.63 ± 25.03 vs. 0.00 ± 0.00; *p* = 0.027) (Figure 1). Furthermore, Group A patients showed statistically significant higher scores of RSI (23.35 ± 5.20 vs. 3.60 ± 0.34; *p* = 0.0001), SISS (1.25 ± 0.91 vs. 0.30 ± 0.48; *p* = 0.001), BEWE (1.60 ± 0.99 vs. 0.10 ± 0.31; *p* < 0.001) and VAS (3.45 ± 2.64 vs. 0.00 ± 0.00; *p* < 0.001). In addition, Group A patients showed higher values of xerostomia (70% vs. 20%; *p* = 0.013), halitosis (55% vs. 10%; *p* = 0.021), dysgeusia (40% vs. 0%; *p* = 0.022) and hyperesthesia (75% vs. 20%; *p* = 0.006). Mean salivary pH of Group A and Group B were 6.8 ± 0.89 and 7 ± 0.47, respectively. Table 2 summarizes these results. Oral pepsin levels correlated linearly with RSI scores (*p* = 0.028) (Figure 2). Subgroup A PEP + patients, compared to group B and subgroup A PEP − patients, showed higher incidence of LPR (100% vs. 44.6%; *p* = 0.001), lower salivary flow (0.60 ± 0.40 vs. 1.25 ± 0.96; *p* = 0.039), higher SISS scores (1.42 ± 0.90 vs. 0.61 ± 0.77; *p* = 0.019), higher BEWE scores (1.67 ± 1.07 vs. 0.72 ± 0.96; *p* = 0.022) and higher VAS scores (3.67 ± 2.77 vs. 1.39 ± 2.30; *p* = 0.021). Subgroup A PEP + patients also showed higher halitosis (66.7% vs. 22.2%; *p* = 0.020), dysgeusia (50.0% vs. 11.1%; *p* = 0.027), hyperesthesia (83.3% vs. 38.9%; *p* = 0.019), aphthosis (33.3% vs. 0%; *p* = 0%; *p* = 0.018) and hyperemia of the soft palate (41.7% vs. 0%; *p* = 0.006). Among Group A patients, subgroup A PEP + showed a statistically significant higher incidence of soft palate erythema compared with subgroup A PEP − (41.7% vs. 0%; *p* = 0.050). No other statistically significant differences were found between the latter two groups in relation to the other oral manifestations (Table 3). 

### 3.3. Salivary Flow Rate Results

**Figure 3:**
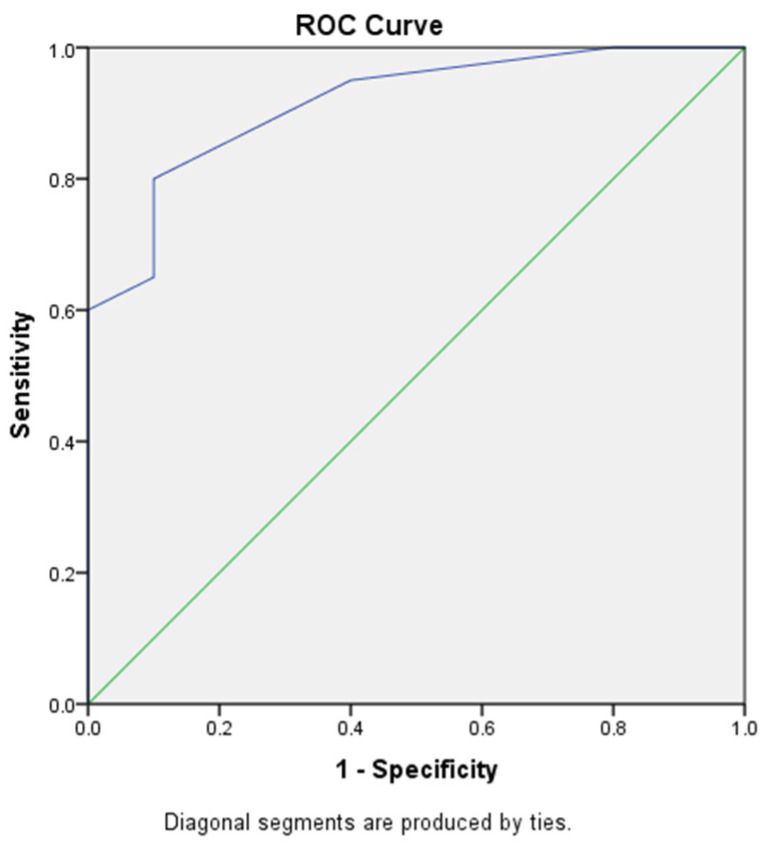
Salivary flow demonstrated excellent accuracy in identifying Group A membership, with an AUC of 0.915.

**Figure 4:**
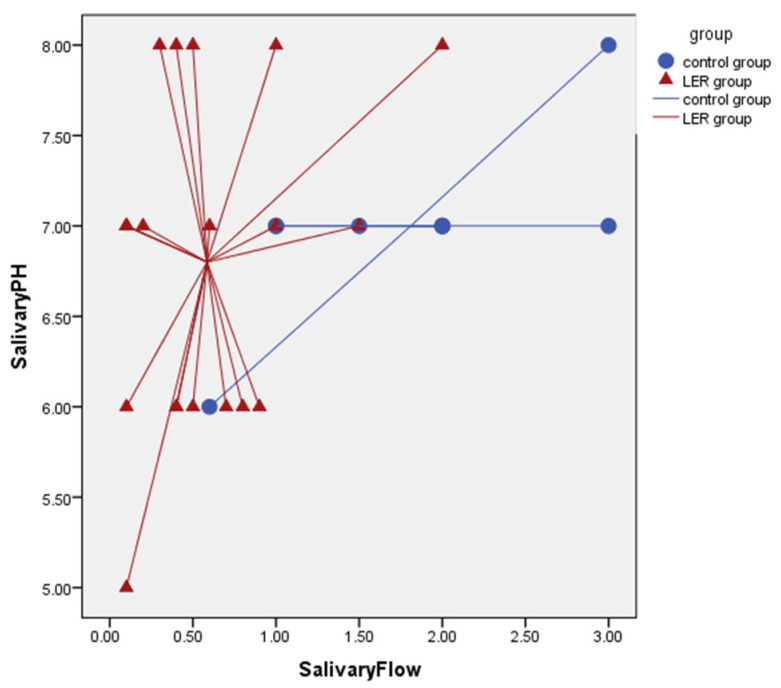
Salivary flow and salivary pH have demonstrated an ability to discriminate healthy patients from those with LPR in 80 of cases.

Mean uSFR of Group A was 0.58 ± 0.5, while mean uSFR of Group B was 1.81 ± 0.80. Salivary flow demonstrated excellent accuracy in identifying Group A membership with an AUC of 0.915 ((0.814–1.000); *p* = 0.0001) (Figure 3). Salivary flow cut-off of <0.1 mL/min was shown to have 100% specificity and 100% PPV, although sensitivity and NPV were low (Table 4). Increased salivary flow was found to be a statistically significant protective factor from having laryngeal reflux with an ODDS of 0.072 ([0.012–0.472]; *p* = 0.072) (Table 5). Salivary flow and salivary pH have finally demonstrated an ability to discriminate healthy patients from those with LPR in approximately 80% of cases (Cross Validate Value 83.3%) (Figure 4).

## 4. Discussion

LPR incidence has substantially increased in the last years due to the growing awareness of this relatively new nosological entity and to the new diagnostic measurements [16]. Notably, although its exact prevalence is still unknown, between 10–30% of patients consulting in ENT complain symptoms referable to LPR [17]. As a matter of fact, the retrograde movement of the gastric content into the upper aerodigestive tract causes an inflammation of the mucous membrane of these areas, which is responsible for the many symptoms reported by the patients, such as hoarseness, excess throat mucus, sensation of a foreign body, chronic cough, postnasal drip and dysphagia [18]. These symptoms reflect the mucosal damage, since the laryngeal and pharyngeal epithelia are far more susceptible than the esophageal epithelium to pepsin injury in the presence of acid, due to the absence of mechanisms of acid elimination. 

### 4.1. The Correlation between GER and Dental Disorders

Recently, several studies have shown that GER could be involved in the development of dental disorders. In particular, the repeated or prolonged exposure of teeth to acid can lead to the dissolution of specific components of the tooth surface, causing loss of tooth substance, caries, hypersensitivity, functional impairment and even tooth fracture. The severity of dental disorders is directly related to the duration of GER, to the pH and type of acid of the refluxed material and the quality and quantity of saliva [19]. Although the pathophysiological mechanisms underlying the development of dental disorders related to reflux are still poorly understood, many hypotheses have been proposed, including the reduction of the salivary buffering capacity or the modification of the pharyngeal/oral microbiota [20].

### 4.2. The Role of LPR in the Development of Dental Disorders

Since LPR is often considered a manifestation of GER disease, it has been questioned whether LPR was also associated with salivary changings and, thus, dental disorders. As shown in the results, the intraoral examination of patients suffering from LPR revealed a higher incidence of dental disorders compared with the control group. Moreover, mean SISS and BEWE scores of Group A patients were statistically significantly higher compared with Group B patients. However, when comparing the PEP + subgroup A with the PEP − subgroup A, no statistically significant difference was found between the SISS, BEWE and DMFT scores. Indeed, subgroup A PEP −, characterized by lower levels of salivary pepsin, had higher values of DMFT, which, albeit not statistically significant, reflected a worse dental status. Only soft palate erythema had a statistically significant higher incidence in subgroup A PEP + compared to subgroup A PEP −. Therefore, pepsin does not appear to be directly correlated with dental erosion, despite the important role it plays in the pathophysiology of mucosal damage [21]. Conversely, salivary changes related to LPR could be associated with dental erosions and caries. In fact, saliva is one of the major elements responsible for homeostasis in the oral cavity and in the digestive tract. Alterations in pH and salivary flow have already been shown to facilitate dental impairment in patients with GER. In addition, spontaneous activation of lymphocyte proliferation, increased phagocytic activity of neutrophils, increased levels or circulating immune complexes and proinflammatory cytokines and lower concentration of secretory IgA have also been demonstrated in patients with GER and concomitant dental caries [22]. As highlighted in the results, even patients suffering from LPR had statistically significantly lower uSFR scores compared to control group. Precisely the reduction of both salivary flow and salivary buffering capacity could cause ulcers, burning mouth, candidiasis, caries and susceptibility to dental erosion, as well as the alteration in the oral microbiome [23]. In this context, salivary flow appears to be a useful parameter in the diagnostic process, where it has demonstrated excellent accuracy in identifying patients suffering from LRP. Indeed, the combined assessment of uSFR and salivary pH allows discrimination of healthy patients from those with LPR. 

The main limitation of this study is the small sample size, which is mainly related to the PEP test kits available. Another limitation could be the use of subjective measures for assessing dental disorders. However, to reduce this bias, multiple rating scales were used, including SISS, BEWE and DMFT, combined with objective investigations, such as the PEP test.

### 4.3. Preventive Measures for Dental Disorders

In light of these results, the finding of demineralization and dental caries on intraoral evaluation must raise the suspicion of LRP, especially in patients who complain of dry mouth, hoarseness, pharyngeal globus, chronic cough and dysphagia, since alterations in salivary volume and oral microbiota due to the reflux of gastroduodenal content in the oropharynx and larynx make the teeth more susceptible to erosion and wear. An early detection of enamel demineralization can prevent the damage from becoming irreversible. Indeed, the enamel structure can be remineralized using specific oral regimens and preventive changes in diet and behavior. The treatment of oral dryness induced by reduced volume of salivary flow may be indicated in patients suffering from LPR, together with Proton Pump Inhibitors (PPIs) and alginates, to improve salivary buffering capacity and prevent associated mucosal and dental damage [24,25].

## 5. Conclusions

The finding of caries and dental disorders in patients who complain of symptoms related to reflux disease must lead to the suspicion of LRP. Early diagnosis and specific treatments are mandatory in order to preserve the buffering capacity and salivary pH and, thus, prevent mucosal and dental damage. 

Further studies on a larger scale are needed to better clarify the role of salivary changes in the etiopathogenesis of dental disorders associated with LPR, in order to correctly guide therapeutic strategies.

## Figures and Tables

**Table 1:** Reflux symptom index (RSI).

Reflux Symptom Index (RSI)						
Within the last month, how did the following problem affect you?	0 = No problem to 5 = Severe problem					
1. Hoarseness or a problem with your voice	0	1	2	3	4	5
2. Clearing your throat	0	1	2	3	4	5
3. Excess throat mucous or postnasal drip	0	1	2	3	4	5
4. Difficulty swallowing food, liquids, or pills	0	1	2	3	4	5
5. Coughing after you ate or after lying down	0	1	2	3	4	5
6. Breathing difficulties or choking episodes	0	1	2	3	4	5
7. Troublesome or annoying cough	0	1	2	3	4	5
8. Sensations of something sticking in your throat or a lump in your throat	0	1	2	3	4	5
9. Heartburn, chest pain, indigestion, or stomach acid coming up	0	1	2	3	4	5

**Table 2:** Summary of the results, comparing Group A with Group B.

	Group A (N = 20) M ± DS or n (%)	Group B (N = 10) M ± DS or n (%)	*p* Value
*Sex, M*	5 (25)	1 (10)	0.326
*Age*	44.90 ± 0.34	31.90 ± 17.11	0.061
*RSI*	23.35 ± 5.20	3.60 ± 0.34	**0.0001**
*Salivary pH*	6.80 ± 0.89	7.00 ± 0.47	0.515
*uSFR*	0.580 ± 0.50	1.81 ± 0.80	**0.001**
*Pepsin*	18.63 ± 25.03	0.00 ± 0.00	**0.027**
*SISS*	1.25 ± 0.91	0.30 ± 0.48	**0.001**
*BEWE*	1.60 ± 0.99	0.10 ± 0.31	**0.0001**
*DMFT*	8.70 ± 3.73	6.00 ± 3.97	0.091
*VAS*	3.45 ± 2.64	0.00 ± 0.00	**0.0001**
*Burning mouth*	4 (20)	0 (0)	0.177
*Xerostomia*	14 (70)	2 (20)	**0.013**
*Sialorrhea*	5 (25)	0 (0)	0.104
*Halitosis*	11 (55)	1 (10)	**0.021**
*Dysgeusia*	8 (40)	0 (0)	**0.022**
*Hyperesthesia*	15 (75)	2 (20)	**0.006**
*Aphthosis*	4 (20)	0 (0)	0.177
*Soft palate erythema*	5 (25)	0 (0)	0.109
*Glossitis*	6 (30)	0 (0)	0.065

**Table 3:** Comparison between subgroup A PEP + and subgroup A PEP −.

	Group A PEP + (N = 12) M ± DS or n (%)	Group A PEP − (N = 8) M ± DS or n (%)	*p* Value
*Sex, M*	3 (25)	2 (25)	0.693
*Age*	40.58 ± 17.34	51.38 ± 11.25	0.139
*RSI*	23.67 ± 6.05	22.87± 3.94	0.727
*Salivary pH*	6.58 ± 0.90	7.12 ± 0.83	0.187
*uSFR*	0.60 ± 0.40	0.55 ± 0.66	0.827
*SISS*	1.42 ± 0.90	1.00 ± 0.92	0.335
*BEWE*	1.67± 1.07	1.50 ± 0.92	0.716
*DMFT*	7.50 ± 3.00	10.50 ± 4.17	0.106
*VAS*	3.67 ± 2.77	3.13 ± 2.58	0.542
*Burning mouth*	3 (25)	1(12.5)	0.465
*Xerostomia*	8 (66.7)	6 (75.5)	0.545
*Sialorrhea*	4 (33.3)	1(12.5)	0.307
*Halitosis*	8 (66.7)	3 (37.5)	0.205
*Dysgeusia*	6 (50.0)	2 (25.0)	0.260
*Hyperesthesia*	10 (83.3)	5 (62.5)	0.295
*Aphthosis*	4 (33.3)	0 (0)	0.102
*Soft palate erythema*	5 (41.7)	0 (0)	**0.050**
*Glossitis*	4 (33.3)	2 (25.0)	0.545

**Table 4:** Specificity, sensitivity, PPV and NPV of uSFR cut-off of <0.1 mL/min.

	Sensitivity	Specificity	PPV	NPV
*Cutoff < 0.1*	25.00%	100.00%	100.00%	40.00%

**Table 5:** Increased salivary flow was found to be a statistically significant protective factor from having laryngeal reflux.

	ODD	CI 95%	*p* Value
*Salivary Flow*	0.072	0.012–0.472	0.004

## Data Availability

The data that support the findings of this study are available from the corresponding author, G.R., upon reasonable request.
